# Prevalence and factors associated with lumbopelvic pain among pregnant women in their third trimester: a cross-sectional study

**DOI:** 10.11604/pamj.2023.46.68.28293

**Published:** 2023-10-25

**Authors:** Samuel Kizito, Milton Wamboko Musaba, Julius Wandabwa, Paul Kiondo

**Affiliations:** 1 Makerere University College of Health Sciences, Kampala, Uganda,; 2 Busitma University, Faculty of Health Sciences, Tororo, Uganda

**Keywords:** Pregnancy-related lumbopelvic pain, pregnancy, Uganda

## Abstract

**Introduction:**

pregnancy related lumbopelvic pain, which refers to low back pain and pelvic girdle pain, is a common musculoskeletal disorder affecting quality of life. The purpose of this study was to establish the prevalence and the factors associated with lumbopelvic pain among pregnant women in their third trimester

**Methods:**

four hundred and nineteen pregnant women were included in this institutional-based cross-sectional study. The study was carried out from October 2018 to March 2019 at Kawempe national referral hospital in Uganda. Pregnant women in the third trimester participated in the study. Pregnant women with preexisting backache, a fracture or surgery to the back, hip or pelvic area in the preceding 12 months were excluded. Lumbopelvic pain was defined as low back pain and pelvic girdle pain. Bivariate and multivariable logistic regression were carried out to establish the factors associated with lumbopelvic pain. The presence of lumbopelvic pain was assessed for and diagnosed using the illustrations in the pelvic girdle questionnaire.

**Results:**

the prevalence of pregnancy related lumbopelvic pain was 46% (95% CI: 40.8-50.4). Most women who had pregnancy related lumbopelvic pain experienced lumbar pain. The factors independently associated with pregnancy related lumbopelvic pain (PLPP) were being HIV sero positive [adjusted odds ratio (AOR) 2.25, 95% CI: 1.17-4.35] and having no monthly income (AOR 0.53, 95% CI: 0.30-0.94).

**Conclusion:**

in this study, PLPP is common in women attending antenatal clinic in their third trimester. The factors associated with PLPP were being HIV positive and having no income. In future pregnant women who come for antenatal care with pregnancy related lumbopelvic pain should be given appropriate advice and support.

## Introduction

Pregnancy-related lumbopelvic pain (PLPP) is a common musculoskeletal disorder in pregnancy [1,2]. It refers to low-back pain and pelvic girdle pain which has a negative effect on a woman´s wellbeing. Pelvic girdle pain (PGP) is the pain felt between the posterior iliac crest and the gluteal fold, close to the sacroiliac joints, and may radiate to the anterior aspect of the thigh. Pubic symphysis pain may occur in association or alone and may radiate to the anterior aspect of the thigh [3,4]. Low back pain (LBP) is pain felt between the 12^th^ vertebrae and gluteal fold and may or may not radiate to the lower limb [5]. The prevalence of PLPP is from 20% to 80% with most studies reporting a prevalence of 50% [6-9]. The differences in the reported prevalence are attributed to the terminology, definitions and methods used in the different studies. The pain is progressive and increases as pregnancy advances [5,10]. The pain starts at 18^th^ gestational week and peaks between the 24^th^ and 36^th^ weeks [4,10]. Sometimes the pain begins in the first trimester and may persist up to the postpartum period or persist and become permanent [4,11]. Recurrence of PLPP in a subsequent pregnancy is common [3,8]. Pregnancy related lumbopelvic pain is not life-threatening, however, many women experience severe discomfort that may interfere with their daily activities such as walking, productivity, employment and even sleeping thus reducing the women´s quality of life [12,13]. This has economic implications for women as they lose productive life [14]. Absenteeism is common and is related to the intensity of pain and the degree of disability [13,14]. Current theories about the development of PLPP include increased mobility of the joints due to the effect of relaxin hormone on collagen, weight gain, weight of the growing fetus, fatigue and the enormous load.

The physical load leads to a change in posture, an increase in the lumbar lordosis, increased pelvic tilt and overstraining of abdominal muscles [15,16]. The risk factors for PLPP include a history of lumbopelvic pain in a previous pregnancy, smoking, oral contraceptive use, strenuous work, advanced maternal age, pain during menstruation, parity and prolonged second stage of labour [10,17]. Worldwide, PLPP is a common problem, although many women receive no treatment. They are counselled that the condition is temporary and self-limiting [14]. Yet negative psychological effects and disability have been reported [10,18]. Other women have requested an elective caesarean section or induction of labour so as to alleviate the pain to the detriment of both the mother and the baby [17,19]. The risk factors for PLPP include a history of lumbopelvic pain in a previous pregnancy, smoking, oral contraceptive use, strenuous work, advanced maternal age, pain during menstruation, parity and prolonged second stage of labour [10,17]. There are few guidelines for the management of worldwide PLPP may be due to the belief that the condition is not a serious health problem to the mother and her unborn baby [17,20]. Some health workers hold the argument that PLPP is a normal process of pregnancy that should not be acknowledged as a specific pain condition with debilitating effects on the health of pregnant women [3,20]. The prevalence of pregnancy-related lumbopelvic pain in Ugandan women is not known. The aim of this study was to establish the prevalence and factors associated with lumbopelvic pain among pregnant women in their third trimester attending an antenatal clinic in a public hospital in Uganda.

## Methods

**Design:** this was a cross-sectional study carried out from October 2018 to March 2019.

**Setting:** the study was carried out in the antenatal clinic at Kawempe Hospital. Kawempe Hospital is a National referral hospital in Uganda and a teaching hospital for Makerere University College of Health Sciences. On average 9,600 mothers attend antenatal clinic at the hospital every month.

**Study population:** the study population consisted of pregnant women who had come to attend an antenatal clinic at Kawempe Hospital. The women included in the study were in their third trimester of gestation (from 28 weeks) with a singleton pregnancy. Women were excluded if they had preexisting backache unrelated to pregnancy, a fracture, or surgery to the back, hip or pelvic area in the preceding 12 months.

**Sample size calculation:** the sample size was calculated using a formula for comparing two proportions [21]. We assumed the risk of getting PLPP to be 15% in obese women and 5% in women of normal weight as was found in a study in Spain [8]. A sample size of 419 mothers was found to be adequate with a power of 80% and a confidence interval of 95%.

**Variables:** our primary outcome was pregnancy-related lumbopelvic pain diagnosed using the illustration in the pelvic girdle questionnaire [22]. Women who reported pain were asked to locate the pain on the illustration in the pelvic girdle questionnaire. They were then asked to point to the site of pain in their body. If the women pointed to the lower back, they were taken to have lower back pain. If they pointed to the pelvis, they were considered to have pelvic girdle pain. If the women pointed to the lower back and pelvis, they were considered to have lower back pain and pelvis girdle pain [5,22]. A reliable and valid pelvic girdle questionnaire for the French-Canadian population was used [22]. The tool was pretested on a sample of pregnant women before the beginning of the study. Women were asked if they had PLPP in the last seven days or if they were having the pain currently and if they had pain in the previous pregnancy. The women were asked to rate the pain using a 10-point visual analog scale where a score of 0 was for no pain while a score of 10 was for very severe pain [23]. The independent variables were the sociodemographic characteristics (maternal age in completed years, weight in kilograms, height in meters, body mass index, marital status, occupation and level of education), medical and obstetric factors (gestational age estimated from the first day of the last normal period, parity, mode of delivery in a previous pregnancy, interpregnancy interval and antenatal attendance) and personal factors like previous history of lumbopelvic pain.

**Data collection procedures:** data was collected using an interviewer-administered questionnaire. The data was collected by two research assistants who were qualified midwives. The research assistants approached the women who had come to attend the antenatal clinic and gave them information about the study. The women who accepted to join the study were checked for eligibility. Using systematic sampling every third woman was selected and taken through an informed consent procedure and, recruited in the study. The information was collected from the participants.

**Data management and statistical analysis:** the data collected were cleaned coded and entered by two data entry clerks in EPIDATA 3.1 software package and imported to STATA version 13 for analysis. The prevalence of lumbopelvic pain was calculated as a proportion of women who had lumbopelvic pain over the total number of participants in the study. The categorical data were summarized as percentages and continuous variables were summarized as means and standard deviations. To assess factors associated with lumbopelvic pain, a bivariate analysis was done. The percentages at each level of exposure were presented and compared using the Chi-square test. Multivariable analysis was done to determine the factors that were independently associated PLPP. Factors that had a p-value of 0.2 or less and factors that were known a priori to be associated with PLPP were entered into a multivariable logistic regression model and adjusted. The backward elimination method was used. Results are presented as adjusted odds ratios with their corresponding 95% confidence intervals.

**Ethical considerations:** Institutional approval for the study was obtained from the Makerere University School of Medicine Research and Ethics Committee (REC-REF 2019-018), Kawempe Hospital Research and Ethics Committee, and the National Council for Science and Technology in Uganda. The participants in this study were given information about the study and they gave written informed consent. Permission was obtained from the Institutional review boards to study participants below 18 years of age and assent was obtained from the participants. Only participant's study numbers were used no names were entered in the database.

## Results

**Prevalence of pregnancy-related lumbopelvic pain among women in their third trimester:** the proportion of women with pregnancy-related lumbopelvic pain among 419 study participants recruited in the study was 191/419. This gave a prevalence of prevalence of 46% (95 CI: 40.8-50.4).

**Socio-demographic and socio-economic characteristics of study participants:** the socio-demographic characteristics of the 419 participants included in the study are shown in Table 1. The mean age of the participants was 26.4 (SD ±5.0) with range of 17 to 42 years. Twenty-six percent (112/419) of the women were overweight or obese. More than half the women had secondary education or above however, 17% of the women had no monthly income. Most women could take any breaks at work and one in ten of the women were HIV seropositive. The rest of the details are in Table 1.

**Table 1:** socio-demographic characteristics of study participants

Characteristics	Frequency (n=419)	Percentage (%)
**Age in years**		
<20	35	8
20-30	314	75
>30	70	17
**Address**		
Kampala	264	63
Wakiso and others	155	37
**BMI**		
Underweight	36	9
Normal weight	271	65
Overweight	112	27
**Marital status**		
Single	19	5
Married	400	95
**Level of education**		
Primary and below	135	32
Secondary and tertiary	284	68
**HIV status**		
Positive	50	12
Negative	369	88
**Family size (No of persons)**		
Two	104	25
3-5	201	48
6-10	114	27
**Occupation**		
Housewife	267	64
Student/unemployed	6	1
Self-employed	107	26
Others (Employed)	39	9
**Able to take any breaks at work**		
Yes	416	99
No	3	1
**Monthly income (UGX)**		
No income	73	17
≥300,000	73	17
>300,000	273	66

**Obstetric characteristics of the study participants:** the obstetric characteristics of the participants are shown in Table 2 below. Thirty percent of the participants were nulliparous. Most participants had no pain prior to pregnancy. Most women who had PLPP, had lumbar pain. Four percent of the participants had pain that had persisted for more than one month and 22 of the participants had very severe pain.

**Table 2:** obstetric characteristics of study participants

Characteristics	Frequency (n=419)	Percentage (%)
**Antenatal care during pregnancy**		
Less than four visits	351	84
Four or more visits	68	16
**Gravidity**		
One	124	30
Two- four	251	60
Five or more	44	10
**Lumbopelvic pain prior to pregnancy**		
Yes	18	4
No	401	96
**Type pregnancy related lumbopelvic pain during pregnancy**		
No pain	191	46
Lumbar pain	124	30
Pelvic girdle pain	82	19
Mixed	22	5
**Pain duration during pregnancy**		
No pain	191	46
< 1 week	37	9
1 week-1 month	174	41
>1 month	17	4
**Pain severity**		
No pain	191	46
Mild pain	126	30
Moderate pain	80	19
Severe pain	22	5
**Parity**		
Nulliparous	127	30
1-2	214	51
≥3	78	19
**Mode of delivery**		
Vaginal delivery	200	48
Caesarean delivery	45	11
Both	47	11
N/A for nulliparous	127	30
**Birth interval**		
< 1 year	15	4
1-2 years	255	61
>2 years	22	5
N/A for nulliparous	127	30

**Factors associated with pregnancy-related lumbopelvic pain among pregnant women:** the factors associated with pregnancy-related lumbopelvic pain among the 419 women in their third trimester are shown in Table 3. The factors associated with PLPP were being HIV positive, attending the antenatal clinic for the second time, and being gravida 2.

**Table 3:** bivariate analysis of socio-demographic and socio-economic factors associated with pregnancy-related lumbopelvic pain

Characteristics	Experienced pain (n=191)	Never had pain (n=228)	OR (95% CI)	P-value*
**Age in years (Mean±SD)**	**(26.4±5.12)**	**(26.4±4.98)**		
<20	15 (8)	20 (9)	1.00	
20-30	140 (73)	174 (76)	0.93 (0.46-1.89)	0.845
>30	36 (19)	34 (15)	0.71 (0.31 – 1.60)	0.408
**Address**				
Kampala	119 (62)	145 (64)	1.06 (0.71-1.57)	0.785
Wakiso and others 1	72 (38)	83 (36)	1.00	
**BMI**				
Underweight	16 (8)	20 (9)	0.98 (0.48 – 1.97)	0.952
Normal weight	119 (62)	152 (66)	1.00	0.952
Overweight	56 (29)	56 (25)	0.78 (0.50 – 1.22)	0.277
**Marital status**				
Single	12 (6)	7 (3)	1.00	
Married	179 (94)	221 (97)	2.12 (0.82 – 5.49)	0.123
**Level of education**				
Primary and below	61 (32)	74 (32)	1.00	
Secondary and tertiary	130 (68)	154 (68)	0.98 (0.65 – 1.47)	0.910
**HIV status**				
Positive	15 (8)	35 (15)	2.13 (1.12-4.03)	0.020*
Negative	176 (92)	193 (85)	1.00	
**Family size**				
Two	41 (21)	63 (28)	1.00	
3-5	94 (50)	107 (47)	0.74 (0.46 – 1.20)	0.222
6-10	56 (29)	58 (25)	0.67 (0.39-1.15)	0.151
**Occupation**				
Housewife	127 (66)	140 (61)	1.05 (0.53 – 2.05)	0.893
Student/unemployed	2 (1)	4 (2)	1.90 (0.31 - 11.61)	0.487
Self-employed	43 (23)	64 (28)	1.41 (0.68 – 2.96)	0.357
Others (Employed)	19 (10)	20 (9)	1.00	
**Monthly income (UGX)**				
No income	129 (67)	144 (63)	0.69 (0.41 – 1.18)	0.176
≥300,000	34 (18)	39 (17)	0.71 (0.37 - 1.38)	0.316
>300,000	28 (15)	45 (20)	1.00	
**ANC during pregnancy**				
Less than four times	155 (81)	196 (86)	0.70 (0.42 - 1.18)	0.185
More than four times	36 (19)	32 (14)	1.00	
**Gravidity**				
One	55 (29)	69(30)	1	1
Two-four	114 (60)	137 (60)	0.96 (0.40 – 1.58)	0.846
More than five	22 11.5)	22 (10)	0.80 (0.40 – 1.59)	0.519
**Lumbopelvic pain prior to pregnancy**				
Yes	11 (6)	7 (3)	0.52 (0.20 – 1.36)	0.183
No	180 (94)	221 (97)	1.00	
**Parity**				
Nulliparous	56 (29)	71 (31)	1.20 (0.68 – 2.12)	0.519
1-2	97 (51)	117 (51)	1.15 (0.68 – 1.93)	0.609
≥3	38 (20)	40 (18)	1.00	
**Mode of delivery**				
Vaginal delivery	92 (48)	108 (48)	0.93 (0.59 1.45)	0.736
Caesarean section	17 (9)	28 (12)	1.30 (0.65 - 2.61)	0.462
Both	26 (14)	21 (9)	0.64 (0.32 - 1.25)	0.189
N/A for Nulliparous	56 (29)	71 (31)	1.00	
**Birth interval**				
<1 year	8 (4)	7 (3)	0.69 (0.24 – 2.02)	0.498
1-2 years	116 (61)	139 (61)	0.95 (0.62-1.45)	0.796
>2 years	11 (6)	11 (5)	079 (0.32 – 1.95)	0.608
N/A for nulliparous	56 (29)	71 (31)	1.00	

P-value* for logistics regression, statistically significant values (p-value<0.05), SD: means standard deviation; BMI: body mass index; HIV: human immunodeficiency virus; UGX: Uganda shillings; ANC: antenatal clinic; N/A: not applicable

**Multivariable analysis for the factors associated with pregnancy-related lumbopelvic pain:** the factors that were significantly associated with pregnancy-related lumbopelvic pain among women attending antenatal after controlling for other factors are shown in Table 4. The factors were: HIV-positive women had a 2.13 higher odds (adjusted odds ratio 2.13; 95% CI: 1.12-4.03) of PLPP compared to HIV-negative women and having no monthly income was associated with 31% lower odds (adjusted odds ratio 0.69; 95% CI: 0.41-0.94) of having PLPP compared to women with a who had a monthly income of more than 300,000 Uganda shillings a month.

**Table 4:** multivariate analysis of factors associated with lumbopelvic pain

Characteristics	Odds ratios	95% CI	P-value*
**Age in years (Mean ± SD)**			
<20	1.00		
20-30	0.84	0.40 – 1.76	0.646
>30	0.70	0.28 – 1.77	0.451
**BMI**			
Under weight	2.79	0.98 - 7.92	0.054
Normal weight	1.00		
Overweight	2.68	0.90 - 8.03	0.078
**Marital status**			
Single	1.00		
Married	2.32	0.87 - 6.21	0.094
**HIV status**			
Positive	2.25	1.17 - 4.35	0.015*
Negative	1.00		
**Family size**			
Two	0.69	0.42	0.153
3-5	0.65	0.35	0.174
6-10	1.00		
**Monthly income (UGX)**			
No income	0.53	0.30 - 0.94	0.029*
≥300,000	0.68	0.34 - 1.15	0.27
>300,000	1.00		
**Lumbopelvic pain prior to pregnancy**			
Yes	0.62	0.22 - 1.76	0.375
No	1.00		
**History of abortion**			
Yes	0.90	0.55 - 1.47	0.671
No	1.00		

P-value* for a logistics regression, statistically significant values (p-value<0.05; SD: standard deviation; BMI: body mass index; HIV: human immunodeficiency virus; UGX: Uganda shillings

## Discussion

This study determined the prevalence of and factors associated with pregnancy-related lumbopelvic pain among pregnant women attending antenatal clinics at Kawempe Referral Hospital in Uganda. This prevalence was 46% in Kawempe and was similar to what was found in Australian women where lumbopelvic pain was one of the most common complaints by the women [24]. Although, the prevalence of PLPP in our study was higher than what was found in other studies in Nepal [7] and in Kuwait [25]. The possible explanation for a higher prevalence in our study is that all our participants were in their third trimester of gestation. Increasing gestational age is known to increase the risk of PLPP [5,8,10,26]. The prevalence in the current study was however lower than what was found in other studies [6,8,27] elsewhere. The variation in the prevalence may be due to lack of a universally acceptable classification system for this condition and the different diagnostic criteria used. Some studies use questionnaires or clinical examination while others use a combination of both methods to assess, diagnose, and collect data [6,17,28,29]. Studies carried out in many settings show that pregnancy-related lumbopelvic pain is a global health problem affecting about half of pregnant women [19]. The pain greatly affects the pregnant women´s quality of life [3,13]. However, many women do not receive appropriate management from healthcare workers. A number of healthcare workers regard this as a normal process of pregnancy [19,20,24,30]. This is probably because PLPP is poorly defined without a clear etiology [6,12]. Pregnancy related lumbopelvic pain for some women can be a debilitating pain preventing them from performing their daily chores. In this study, one in four of the women experienced moderate to severe pain. The severity of pain as experienced by women in this study is similar to what has been found elsewhere [3,7,25].

In this study, most participants experienced lumbar pain as opposed to pelvic girdle pain. This is similar to what was found in a study in Kuwait [25] and in Nepal [7] but different from what was found in another study in which pelvic girdle pain was more common [3]. Lumbopelvic pain in pregnancy is due to biomechanical and hormonal mechanisms such as increased body weight, changes in posture with increased lumbar lordosis, increased laxity of the ligaments of the pelvis and the spine, and, increased intra-abdominal and intrauterine pressures [15,25]. About 10% of the women experienced pain in both lumbar and pelvic girdle pain. This is similar to what has been found in studies done elsewhere [7]. The prognosis of PLPP is usually good with most women recovering after delivery although persistent pain has been reported several months or even years after delivery [4,6]. Persistent pain is associated with the severity of PLPP and pain involving all three pelvic joints [4,11]. In this study, women with no monthly income were less likely to have PLPP. This is in contrast to other studies in which there was no association between women´s income and the development of lumbopelvic pain [7,10]. The finding of lower odds of lumbopelvic pain in women with no income in this study was unexpected. However, most women in this study were housewives who were more likely to engage in physical activities. Physical exercise has been shown to reduce lumbopelvic pain [17,19]. In addition, many of the women who seek free services at this facility are from the surrounding slum areas and are mostly of low socioeconomic status. Women who were HIV positive were more likely to complain of pregnancy-related lumbopelvic pain than women who were HIV seronegative. Pain is a common symptom in HIV-infected patients and can be debilitating [31]. The pain can appear in all stages of the disease. Low back pain has been reported and can be a manifestation of acute retroviral syndrome. This can be due to neuropathy or some antiretroviral drugs or some non-HIV-related neuromuscular problems, [31-33]. The use of a pretested tool to assess the pain is a major strength of this study. This study is not without limitations, the cross-sectional design which could not establish the causal effect relationship, and recall bias. In addition, diagnostic tests for pelvic girdle pain which needed the assistance of a trained physiotherapist were not done.

## Conclusion

Pregnancy related lumbopelvic pain is common in pregnant women attending antenatal clinic at Kawempe national referral and teaching hospital. The pain was severe in some women who were forced to miss work. Being HIV seropositive increased the risk of PLPP while women who had no income were at a reduced risk. In future, pregnant women who come for antenatal clinic should routinely be asked about the presence of PLPP and given appropriate advice and support.

### What is known about this topic

- *Pregnancy related lumbopelvic pain is common in pregnancy and affects women’s quality of life*.

### What this study adds

- *Pregnancy related lumbopelvic pain is common among pregnant women attending antenatal clinic at Kawempe hospital in Uganda;*
- *Being HIV positive was associated with an increased risk of pregnancy related lumbopelvic pain*.

## References

[1] Gallo-Padilla D, Gallo-Padilla C, Gallo-Vallejo FJ, Gallo-Vallejo JL (2016). Low back pain during pregnancy, Multidisciplinary approach]. Semergen.

[2] Tan EK, Tan EL (2013). Alterations in physiology and anatomy during pregnancy. Best Pract Res Clin Obstet Gynaecol.

[3] Gutke A, Betten C, Degerskar K, Pousette S, Olsen MF (2015). Treatments for pregnancy-related lumbopelvic pain: a systematic review of physiotherapy modalities. Acta Obstet Gynecol Scand.

[4] Kanakaris NK, Roberts CS, Giannoudis PV (2011). Pregnancy-related pelvic girdle pain: an update. BMC Med.

[5] Lardon E, St-Laurent A, Babineau V, Descarreaux M, Ruchat SM (2018). Lumbopelvic pain, anxiety, physical activity and mode of conception: a prospective cohort study of pregnant women. BMJ Open.

[6] Gutke A, Boissonnault J, Brook G, Stuge B (2018). The Severity and Impact of Pelvic Girdle Pain and Low-Back Pain in Pregnancy: A Multinational Study. J Womens Health (Larchmt).

[7] Shijagurumayum Acharya R, Tveter AT, Grotle M, Eberhard-Gran M, Stuge B (2019). Prevalence and severity of low back-and pelvic girdle pain in pregnant Nepalese women. BMC Pregnancy Childbirth.

[8] Kovacs FM, Garcia E, Royuela A, Gonzalez L, Abraira V, Spanish Back Pain Research N (2012). Prevalence and factors associated with low back pain and pelvic girdle pain during pregnancy: a multicenter study conducted in the Spanish National Health Service. Spine (Phila Pa 1976).

[9] Mens JM, Huis in't Veld YH, Pool-Goudzwaard A (2012). Severity of signs and symptoms in lumbopelvic pain during pregnancy. Man Ther.

[10] Backhausen MG, Bendix JM, Damm P, Tabor A, Hegaard HK (2019). Low back pain intensity among childbearing women and associated predictors. A cohort study. Women Birth.

[11] Bergstrom C, Persson M, Nergard KA, Mogren I (2017). Prevalence and predictors of persistent pelvic girdle pain 12 years postpartum. BMC Musculoskelet Disord.

[12] Mackenzie J, Murray E, Lusher J (2018). Women's experiences of pregnancy related pelvic girdle pain: A systematic review. Midwifery.

[13] Gutke A, Olsson CB, Vollestad N, Oberg B, Wikmar LN, Robinson HS (2014). Association between lumbopelvic pain, disability and sick leave during pregnancy-a comparison of three Scandinavian cohorts. J Rehabil Med.

[14] Lotzke H, Jakobsson M, Brisby H, Gutke A, Hagg O, Smeets R (2016). Use of the PREPARE (PREhabilitation, Physical Activity and exeRcisE) program to improve outcomes after lumbar fusion surgery for severe low back pain: a study protocol of a person-centred randomised controlled trial. BMC Musculoskelet Disord.

[15] Casagrande D, Gugala Z, Clark SM, Lindsey RW (2015). Low Back Pain and Pelvic Girdle Pain in Pregnancy. J Am Acad Orthop Surg.

[16] Elden H, Gutke A, Kjellby-Wendt G, Fagevik-Olsen M, Ostgaard HC (2016). Predictors and consequences of long-term pregnancy-related pelvic girdle pain: a longitudinal follow-up study. BMC Musculoskelet Disord.

[17] Vermani E, Mittal R, Weeks A (2010). Pelvic girdle pain and low back pain in pregnancy: a review. Pain Pract.

[18] Chang HY, Jensen MP, Yang YL, Lee CN, Lai YH (2012). Risk factors of pregnancy-related lumbopelvic pain: a biopsychosocial approach. J Clin Nurs.

[19] Liddle SD, Pennick V (2015). Interventions for preventing and treating low-back and pelvic pain during pregnancy. Cochrane Database Syst Rev.

[20] Pennick V, Liddle SD (2013). Interventions for preventing and treating pelvic and back pain in pregnancy. Cochrane Database Syst Rev.

[21] Schlesselman JJ (1974). Sample size requirements in cohort and case-control studies of disease. Am J Epidemiol.

[22] Girard MP, Marchand AA, Stuge B, Ruchat SM, Descarreaux M (2016). Cross-cultural Adaptation of the Pelvic Girdle Questionnaire for the French-Canadian Population. J Manipulative Physiol Ther.

[23] Ostelo RW, Deyo RA, Stratford P, Waddell G, Croft P, Von Korff M (2008). Interpreting change scores for pain and functional status in low back pain: towards international consensus regarding minimal important change. Spine (Phila Pa 1976).

[24] Pierce H, Homer CS, Dahlen HG, King J (2012). Pregnancy-related lumbopelvic pain: listening to Australian women. Nurs Res Pract.

[25] Al-Sayegh NA, Salem M, Dashti LF, Al-Sharrah S, Kalakh S, Al-Rashidi R (2012). Pregnancy-related lumbopelvic pain: prevalence, risk factors, and profile in Kuwait. Pain Med.

[26] Bjelland EK, Eskild A, Johansen R, Eberhard-Gran M (2010). Pelvic girdle pain in pregnancy: the impact of parity. Am J Obstet Gynecol.

[27] Madeira HG, Garcia JB, Lima MV, Serra HO (2013). [Disability and factors associated with gestational low back pain]. Rev Bras Ginecol Obstet.

[28] Starzec M, Truszczyńska-Baszak A (2017). Pregnancy-related lumbopelvic pain: classifi cationand diagnostics according to European guidelines and a review of literature. Medical Rehabilitation.

[29] Bjelland EK, Stuge B, Engdahl B, Eberhard-Gran M (2013). The effect of emotional distress on persistent pelvic girdle pain after delivery: a longitudinal population study. BJOG.

[30] Gorginzadeh M, Imani F, Safari S (2016). Pregnancy-Related Pelvic Pain: A Neglected Field in Developing Countries. Anesth Pain Med.

[31] Pullen SD, Del Rio C, Brandon D, Colonna A, Denton M, Ina M (2020). Associations between chronic pain, analgesic use and physical therapy among adults living with HIV in Atlanta, Georgia: a retrospective cohort study. AIDS Care.

[32] Merlin JS, Westfall AO, Raper JL, Zinski A, Norton WE, Willig JH (2012). Pain, mood, and substance abuse in HIV: implications for clinic visit utilization, antiretroviral therapy adherence, and virologic failure. J Acquir Immune Defic Syndr.

[33] Molony E, Westfall AO, Perry BA, Tucker R, Ritchie C, Saag M (2014). Low back pain and associated imaging findings among HIV-infected patients referred to an HIV/palliative care clinic. Pain Med.

